# Supplementary material for: Electrostatic interactions guide substrate recognition of the prokaryotic ubiquitin-like protein ligase PafA
Source: Nat Commun. 2023 Aug 29;14:5266. doi: 10.1038/s41467-023-40807-8 (PMC10465538; doi:10.1038/s41467-023-40807-8)
Supplement: Supplementary file 1 — Supplementary Information [file 41467_2023_40807_MOESM1_ESM.pdf]

Electrostatic interactions guide substrate recognition of the prokaryotic ubiquitin-like protein ligase PafA

Matthias F. Block[1], Cyrille L. Delley[1,2], Lena M. L. Keller[1], Timo Stühlinger[1] & Eilika Weber-Ban[1] *

[1] ETH Zurich, Institute of Molecular Biology & Biophysics, CH-8093 Zurich, Switzerland

[2] Current address: University of California, San Francisco

* To whom correspondence should be addressed. E-mail: eilika@mol.biol.ethz.ch

- Supplementary Information -

SUPPLEMENTARY FIGURES

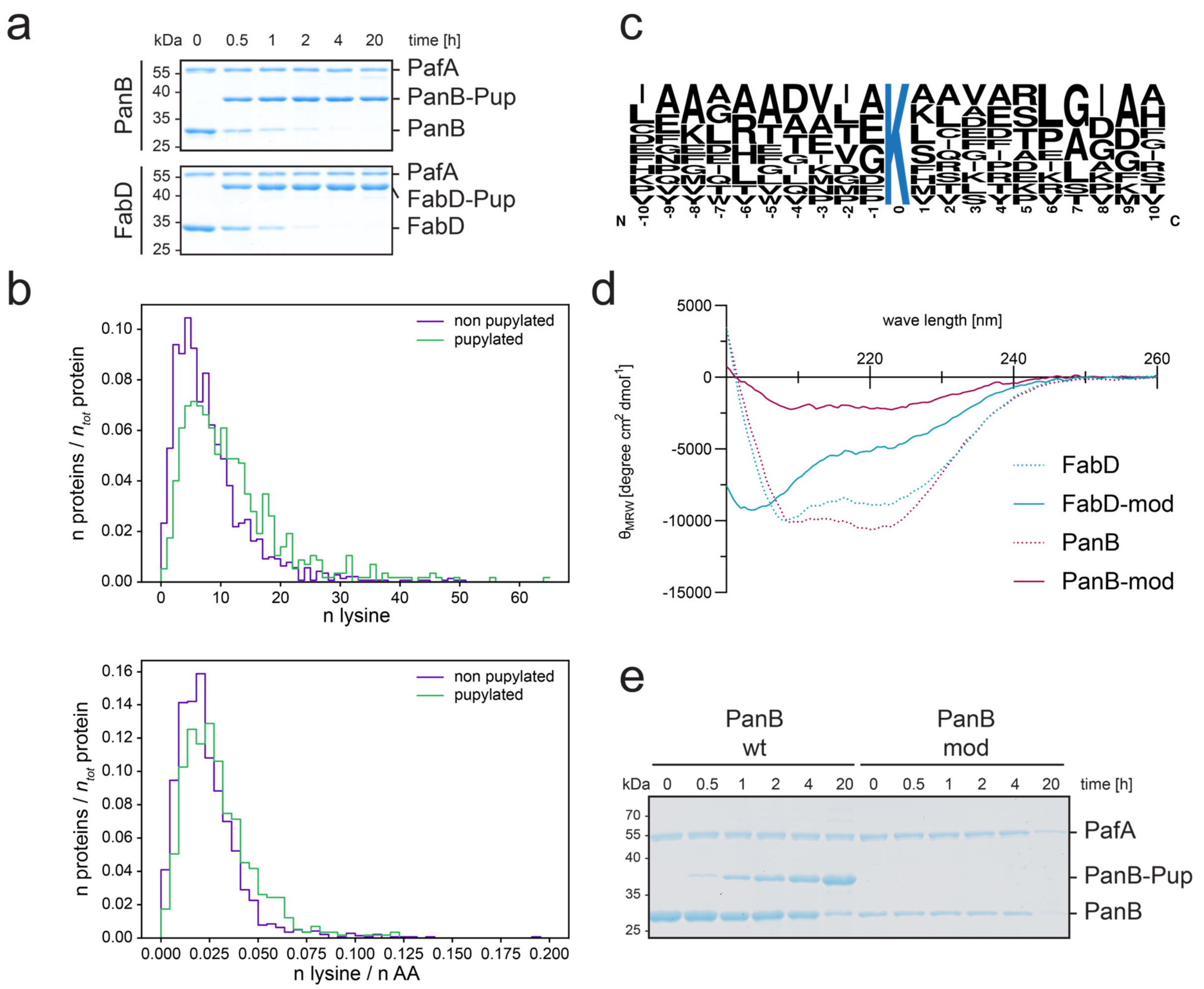

Supplementary Figure 1: Pupylation does not depend on linear sequence context. a Pupylation time course of PanB (top) and FabD (bottom) visualized by SDS-PAGE and Coomassie-staining. Both substrates are pupylated efficiently within 2 h by PafA. Representative gels of three individual experiments are shown. Source Data (uncropped gels) are provided as a Source Data file. **b** Histograms of the total lysine count (top) or lysine density (bottom) plotted against the number of members of the Mtb proteome that have been identified (green) or have not been identified (purple) in pupylome studies. **c** Logo plot of the +/- 10 residues flanking pupylation sites in genuine substrates (see also **Figure 2**, good substrates). The plot was generated using the Berkeley logo plot webserver[1] (last access 08.08.2022). Aligned sequences can be found in Supplementary table 4. **d** Circular dichroism spectra of S-carboxyamidomethylated (mod) FabD and PanB (solid blue and solid purple respectively) and native FabD and PanB (dotted blue and dotted purple respectively). Source Data (raw intensity reads and MRW calculation) are provided as a Source Data file **e** Pupylation time course of native and S-carboxyamidomethylated (mod) PanB visualized by SDS-PAGE and Coomassie-staining. While the native form of PanB was pupylated to 50 % within 4 h and to almost completion within 20 h, modified PanB remained unpupylated within the same time course. Representative gels of three individual experiments are shown. Source Data (uncropped gels) are provided as a Source Data file.

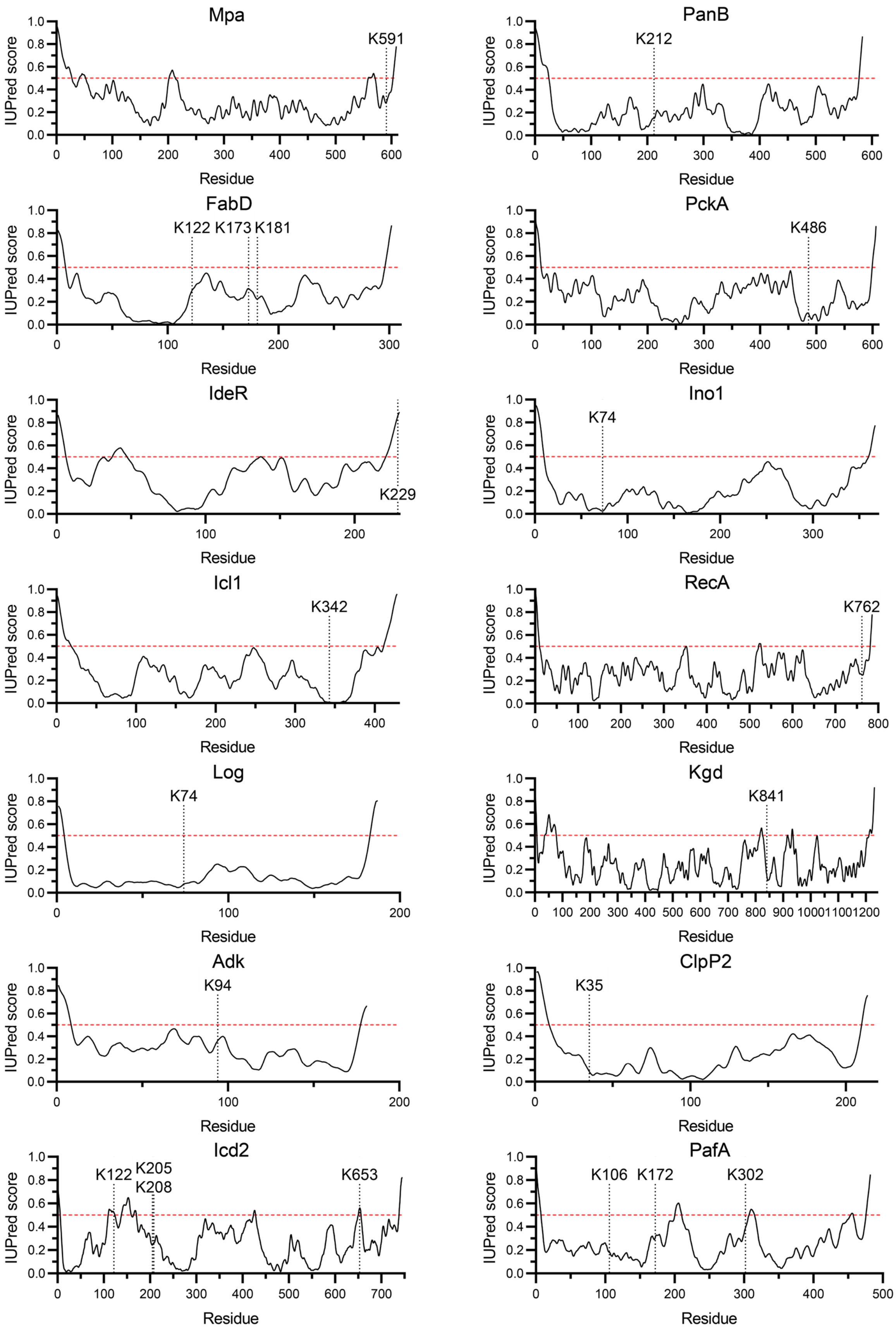
Mpa
K591
PanB
K212
FabD
K122 K173 K181
PckA
K486
IdeR
K229
Ino1
K74
Icl1
K342
RecA
K762
Log
K74
Kgd
K841
Adk
K94
ClpP2
K35
Icd2
K122
K205
K208
K653
PafA
K106 K172 K302
IUPred score
Residue

Supplementary Figure 2: Structural context analysis of pupylation sites in substrates depicted in Fig. 2a. Disorder prediction was performed using the IUPred3 short web server implementation[2] (last access 12.06.2023). Residues with IUPred scores <0.5 were predicted to be in structured regions (below red line), while residues with scores >0.5 were predicted to be in disordered regions (above red line). All lysine residues identified as pupylated in the various proteins, except for IdeR K229 (score 0.86), are predicted to be in in structured regions with IUPred scores <0.5. Source Data (IUPred scores) are provided as a Source Data file

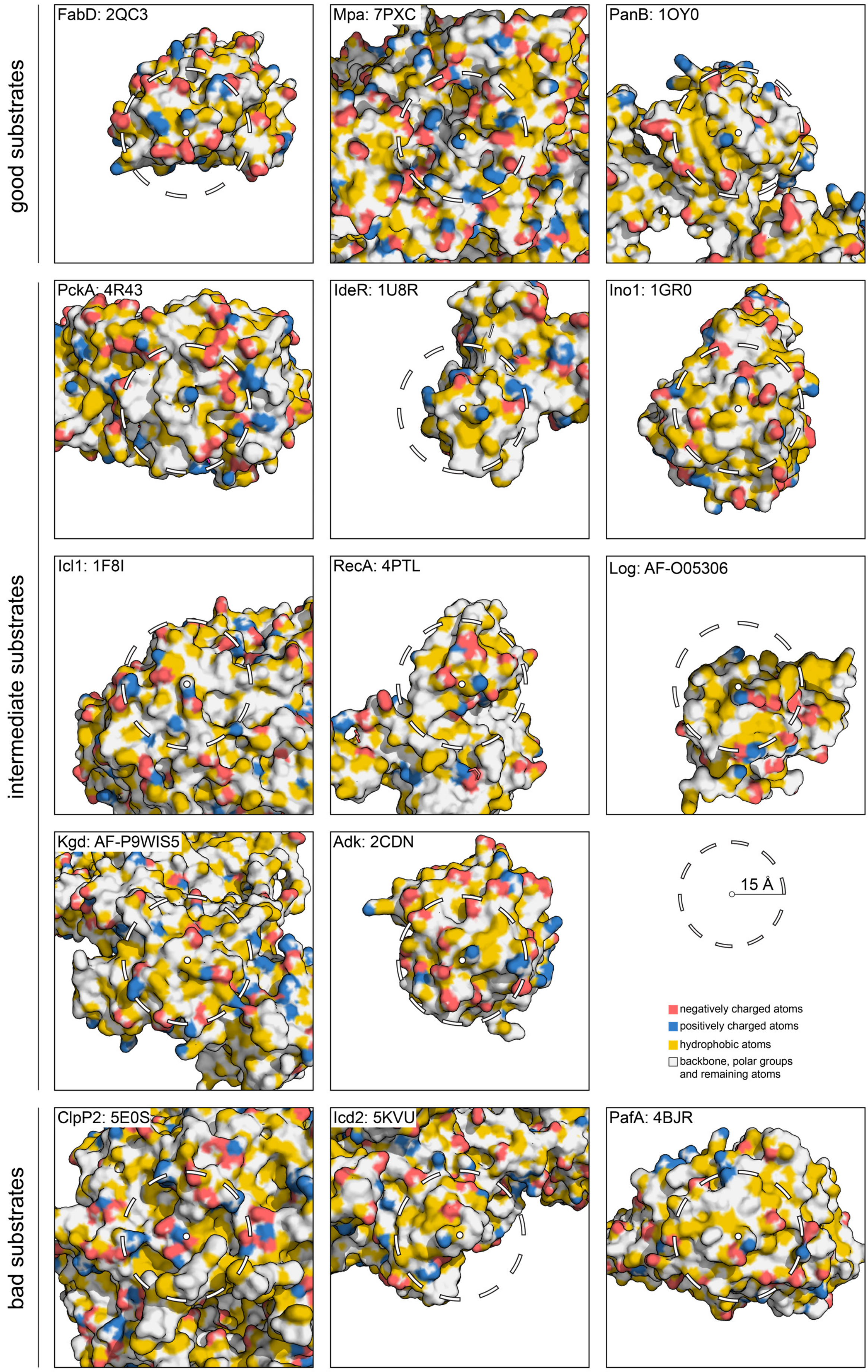
good substrates
FabD: 2QC3
Mpa: 7PXC
PanB: 1OY0
intermediate substrates
PckA: 4R43
IdeR: 1U8R
Ino1: 1GR0
Icl1: 1F8I
RecA: 4PTL
Log: AF-O05306
Kgd: AF-P9WIS5
Adk: 2CDN
15 Å
negatively charged atoms
positively charged atoms
hydrophobic atoms
backbone, polar groups
and remaining atoms
bad substrates
ClpP2: 5E0S
Icd2: 5KVU
PafA: 4BJR

Supplementary Figure 3: Visualization of the protein surface surrounding the pupylation site in the fourteen substrates shown in Fig. 2a. In all substrates of the good and intermediate category, negatively charged, surface-exposed residues are present within a 15 Å radius around the central lysine's C-alpha atom (indicated by the circle and the centered dot). The three substrates from the "bad" category show either a buried lysine (see ClpP2) or only very few negatively charged residues in close proximity to the pupylated lysine.

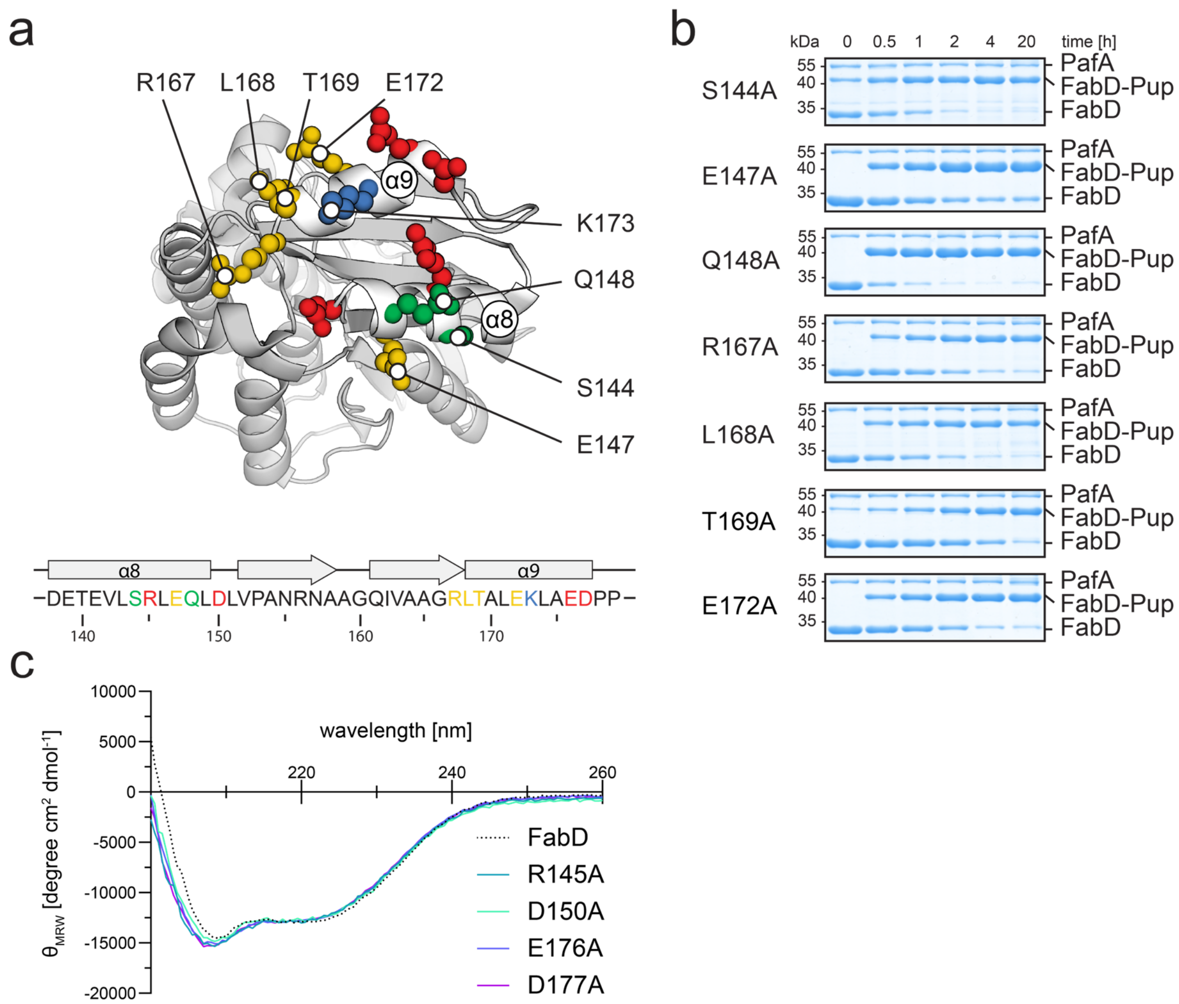

Supplementary Figure 4: Pupylation of FabD is encoded in a three-dimensional recognition motif. a Cartoon representation of FabD analogous to Figure 3a with the remaining mutated residues marked. **b** Pupylation time courses of FabD alanine variants with mild to intermediate effects on pupylation efficiency were followed by SDS-PAGE and Coomassie-staining. Based on the impact on the pupylation time course compared to wild type-like FabD (Figure 3b), contribution of individual residues was mapped in Figure 3a. Representative gels of three individual experiments are shown. Source Data (uncropped gels) are provided as a Source Data file. **c** Circular dichroism spectra of FabD (dotted line) and FabD variants (colored lines) that affected pupylation the strongest. Source Data (raw intensity reads and MRW calculation) are provided as a Source Data file.

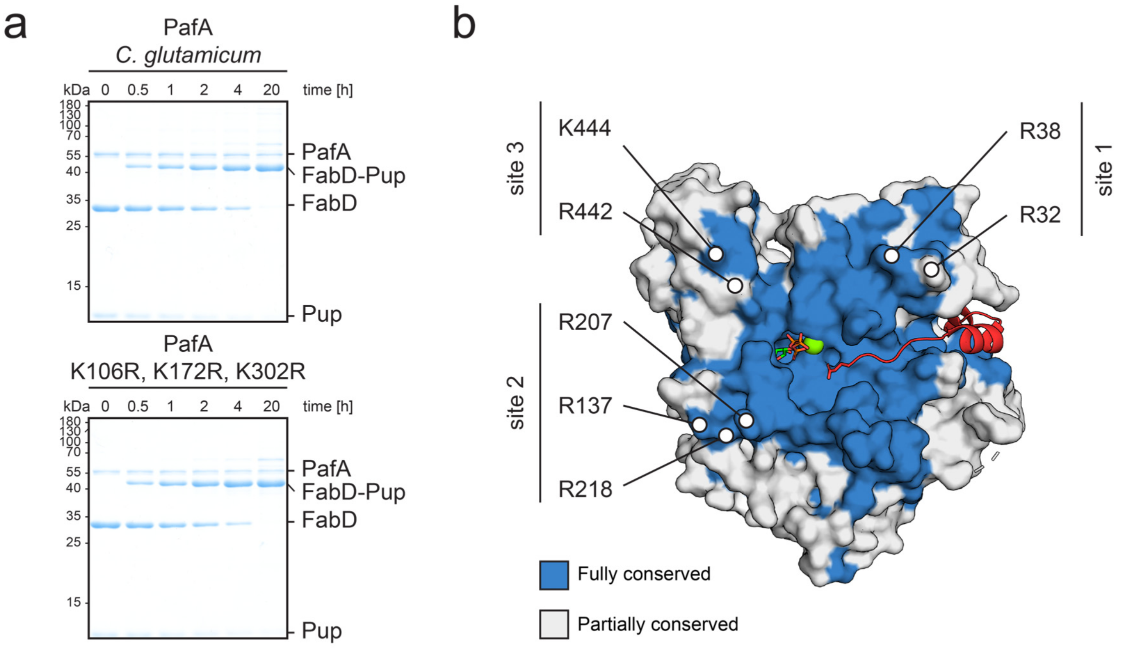

c

```
              10        20        30        40        50        60        70        80
Cgl   MSTVESALTRRIMGIETEYGLTFVDGDSKKLRPDEIARRMFRPIVEKYSSSNIFIPNGSRLYLDVGSHPEYATAECDNLT
Mtb   -------MQRRIMGIETEFGVTCTFHGHRRLSPDEVARYLFRRVVSWGRSSNVFLRNGARLYLDVGSHPEYATAECDSLV
Msm   -------MQRRIMGIETEFGVTCTFHGHRRLSPDEVARYLFRRVVSWGRSSNVFLRNGARLYLDVGSHPEYATAECDNLI
                                     site 1

              90       100       110       120       130       140       150       160
Cgl   QLINFEKAGDVIADRMAVDAEESLAKEDIAGQVYLFKNNVDSVGNSYGCHENYLVGRSMPLKALGKRLMPFLITRQLICG
Mtb   QLVTHDRAGEWVLEDLLVDAEQRLADEGIGGDIYLFKNNTDSAGNSYGCHENYLIVRAGEFSRISDVLLPFLVTRQLICG
Msm   QLVTHDRAGERVLEDLLIDAEQRLADEGIGGDIYLFKNNTDSAGNSYGCHENYLIVRAGEFSRISDVLLPFLVTRQLICGt
                                                          site 2

             170       180       190       200       210       220       230       240
Cgl   AGRIHHPNPLDKGESFPLGYCISQRSDHVWEGVSSATTRSRPIINTRDEPHADSHSYRRLHVIVGDANMAEPSIALKVGS
Mtb   AGKVLQTP-------KAATYCLSQRAEHIWEGVSSATTRSRPIINTRDEPHADAEKYRRLHVIVGDSNMSETTTMLKVGT
Msm   AGKVLQTP-------KAATFCLSQRAEHIWEGVSSATTRSRPIINTRDEPHADAEKYRRLHVIVGDSNMCEATTMLKVGT

             250       260       270       280       290       300       310       320
Cgl   TLLVLEMIEADFGLPSLELANDIASIREISRDATGSTLLSLKDGTTMTALQIQQVVFEHASKWLEQRPEPEFSGTSNTEM
Mtb   AALVLEMIESGVAFRDFSLDNPIRAIREVSHDVTGRRPVRLAGGRQASALDIQREYYTRAVEHLQTR-------EPNAQI
Msm   ASLVLEMIEAGVPFRDFSLDNPIRAIREVSHDLTGRRPVRLAGGRQASALDIQREYYSRAVEYLQSR-------EPNTQI

             330       340       350       360       370       380       390       400
Cgl   ARVLDLWGRMLKAIESGDFSEVDTEIDWVIKKKLIDRFIQRGNLGLDDPKLAQVDLTYHDIRPGRGLFSVLQSRGMIKRW
Mtb   EQVVDLWGRQLDAVESQDFAKVDTEIDWVIKRKLFQRYQDRYDMELSHPKIAQLDLAYHDIKRGRGIFDLLQRKGLAARV
Msm   EQVVDLWGRQLDAVESQDFAKVDTEIDWVIKRKLFQRYQDRYNMELSDPKISQLDLAYHDIKRGRGVFDLLQRKGLAARI

             410       420       430       440       450       460       470       480
Cgl   TTDEAILAAVDTAPDTTRAHLRGRILKAADTLGVPVTVDWMRHKVNRPEPQSVELGDPFSAVNSEVDQLIEYMTVHAESY
Mtb   TTDEEIAEAVDQPPQTTRARLRGEFISAAQEAGRDFTVDWVHLKLNDQAQRTVLCKDPFRAVDERVKRLIASM-------
Msm   TTDEEIDAAVTTPPQTTRAKLRGEFISAAQEAGRDFTVDWVHLKLNDQAQRTVLCKDPFRSVDERVKRLIASM-------
                                                 site 3

Cgl   RS
Mtb   --
Msm   --
```

Supplementary Figure 5: Three positively charged patches on the perimeter of the PafA active site are conserved. a Mutation of lysine residues targeted by self-pupylation in ^{Cgl}PafA does not affect overall pupylation activity of the enzyme. Representative gels of three individual experiments are shown. Source Data (uncropped gels) are provided as a Source Data file. **b** Surface-exposed residues conserved between ^{Cgl}PafA, ^{Mtb}PafA and ^{Msm}PafA are depicted in different colors on the surface representation of the PafA structure (PDB: 4BJR). The conservation mapping was generated using the AL2CO webserver implementation[3] (last accessed 08.08.2022). **c** Sequence alignment of ^{Cgl}PafA, ^{Mtb}PafA and ^{Msm}PafA. Mutated residues for site 1, 2 and 3 are highlighted in red.

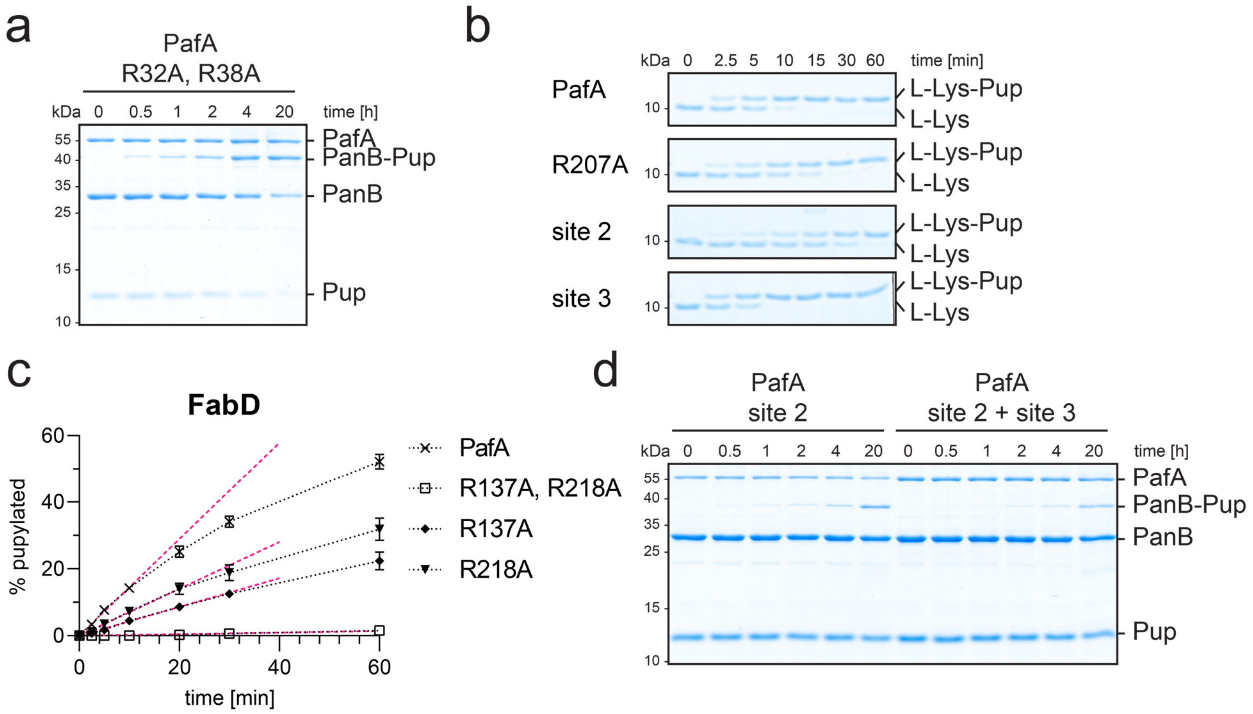

Supplementary Figure 6: Effects of alanine replacement of positively charged residues bordering the PafA active site on *in vitro* pupylation reactions with PanB, FabD and free lysine. a PanB pupylation time course with the site 1 alanine variant of PafA shows that this mutation does not affect the reaction. Representative gels of three individual experiments are shown. Source Data (uncropped gels) are provided as a Source Data file. **b** Pupylation time courses with 0.5 µM of PafA R207A mutant or site 2 and site 3 alanine variants of PafA carried out using L-Lys at close to saturating concentration (100 mM), show that the pupylation reaction itself is only minimally affected by the mutations. Representative gels of three individual experiments are shown. Source Data (uncropped gels) are provided as a Source Data file. **c** Gel densitometric analysis of pupylation assays using the less severe site 2 mutants with FabD as substrate. Apparent initial velocity was determined by linear regression of the first four data points (pink trend line). The fraction of pupylated substrate is expressed in relation to the total amount of substrate present in the reaction. Assays were carried out in independent triplicates, data are presented as mean values with error bars depicting standard deviation. Source Data (raw values from gel densitometric analysis and corresponding uncropped gels) are provided as a Source Data file. **d** Pupylation time courses with the site 2 PafA variant and with a combined site 2/site 3 PafA variant using PanB as substrate, demonstrates that pupylation is not further reduced upon mutation of both sites together. Representative gels of three individual experiments are shown. Source Data (uncropped gels) are provided as a Source Data file.

Supplementary Tables

Supplementary Table 1: Purified proteins tested for *in vitro* pupylation efficiencies. Pupylation reactivity was categorized as explained in the results and in Figures 1c and 2a. ** Log and Pup were excluded from Figure 1b and 1c since they were not detected in the underlying protein abundance dataset[4].

Name	Identifier	PBD	Size [kDa]	Pupylated residue	Pupylation reactivity	Source
Mpa	Rv2115c	7PXC	67.4	591	+++	[5-7]
PanB	Rv2225	1OY0	29.3	212	+++	[5,8,9], this work
FabD	Rv2243	2QC3	30.8	122, 173, 181	+++	[5,6,8], this work
PckA	Rv0211	4R43	67.3	486	++	[5,10,11]
IdeR	Rv2711	1FX7	25.2	229	++	[5,6,12]
Ino1	Rv0046c	1GR0	40.1	73	++	[5,6,11]
Icl1	Rv0467	1F8I	47.1	342	++	[5,6,11]
Log**	Rv1205	AF-O05306-F1	20.0	74	++	[13]
RecA	Rv2737c	4PSK	85.4	762	++	[5,14]
Kgd	Rv1248c	AF-P9WIS5-F1	135.9	841	++	[5,11]
Adk	Rv0733	2CDN	20.1	94	++	[5,6,10], this work
ClpP2	Rv2460c	5E0S	23.5	35	+	[5], this work
Icd2	Rv0066c	5KVU	82.6	122, 205, 208, 653	+	[5,6,10]
Mdh	Rv1240	4TVO	34.3	not identified	+	this work
PafA	Rv2097c	4BJR	51.4	106, 172, 302	+	[5,15], this work
Pup**	Rv2111c	4BJR	6.9	61	+	[5,12]

Supplementary Table 2: Oligonucleotide primers used in this study. Small letters mark overlap with the parental sequence.

Primer name	Sequence (5'→ 3')	Purpose
	PafA point mutations	
PafA_Cgl_K106R_QC	acctgcccagcaatgtcttcACGcgccagcgactcttcg	generation self-pupylation incompetent PafA
PafA_Cgl_K172R_QC	aaggggaaggattcgccACGatccaaaggattggggtgatgg	generation self-pupylation incompetent PafA
PafA_Cgl_K302R_QC	cgctgctccaaccaACGcgaggcatgctcaaag	generation self-pupylation incompetent PafA
PafA_Ala1_long_fw	aaaaagcttGCTccagatgagatagctGCTaggatgtttcgtcccatcgt	mutational screen, PafA site 1
PafA_Ala1_long_rv	ggaatcaccatcaacaaaggtgag	mutational screen, PafA site 1
PafA_Ala2.1_fw	atcaacaccGCTgatgagccacatgcggattcccattcttacGCTaggctgcacgtgat	mutational screen, PafA site 2
PafA_Ala2.1_rv	aatggggcgtgatctagtggtggcacttgatacgccctcccacacgtggtcag	mutational screen, PafA site 2
PafA_Ala2.2_long_fw	accttgtgggtGCTtccatgccgttgaaggcgtt	mutational screen, PafA site 2
PafA_Ala2.2_long_rv	agttttcgtggcagccataagaattgc	mutational screen, PafA site 2
PafA_Ala3_rv	cgacagtcacaggtactcccagagta	mutational screen, PafA site 3
PafA_Ala3_fw2	attggatgGCTcacGCTgtcaaccgac	mutational screen, PafA site 3
PafA_R207A	atcaacaccGCTgatgagccacatgcggattcccattcttaccgcaggctgcacgtgat	mutational screen, PafA; together with PafA_Ala2.1_rv
PafA_R218A	atcaacacccgtgatgagccacatgcggattcccattcttacGCTaggctgcacgtgat	mutational screen, PafA; together with PafA_Ala2.1_rv
PafA_R442A_fw_long	attggatgGCTcacaaagtcaaccgaccggagccaca	mutational screen, PafA; together with PafA_Ala3_rv
PafA_K444A_fw_long	attggatgcgtcacGCTgtcaaccgaccggagccaca	mutational screen, PafA; together with PafA_Ala3_rv
	In vivo complementation	
pafA-upstream fw	gcttaaggctcacatacttcgctgcacaacatcgagct	
pafA-upstrem rv	TTTTCGCCCCGAAGAACGgggcgcccggtcagcgat	
pafA-downstream fw	AAAAGTGCTCATCATTGGAAAAaggtagctgttgtcgcccacg	
pafA-downstream rv	tgttgtgcagcgaagtatgtgagccttaagctgacccggtg	
pafA-RT-PCR-fw	gaggaagacgttggaactgc	
pafA-RT-PCR-rv	tcatgggcatcgagactgaa	
pafA-cPCR1-fw	gagaacgcctcgtcactgggg	
pafA-cPCR1-rv	cggtgcgctgcgcaacgacat	
pafA-cPCR2-fw	tccggccgctcggtgatgtc	
pafA-cPCR2-rv	gaacaccgccgacccgggtc	
pafA-seq1	gaagtacgggaccatgttca	
pafA-seq2	ggccgcttcgtcggccgtca	
pafA-seq3	caccgaggacacctccgccg	
pafA-seq4	cgtcggtcagcagggggacg	
pafA-seq5	agggcgccgcggatca	
LK27_fw		sequencing primer
Msmg_PafA_R193A_fw4	CTgacgaaccgcatgccg	*in vivo* PafA point mutation
Msmg_PafA_R193A_rv4	Ccgtgttgatgatggggcg	*in vivo* PafA point mutation
Msmg_PafA_R204A_fw4	CTcgcctgcacgtcatcg	*in vivo* PafA point mutation
Msmg_PafA_R204A_rv4	Cgtacttctcggcatcggc	*in vivo* PafA point mutation
	Charge swap assay	
PafA_switch2.1_long_fw	atcaacaccGAAgatgagccacatgcggattcccattcttacGAAaggctgcacgtgattgtggg	charge flip, PafA site 2; together with PafA_Ala2.1_rv

PafA_switch2.2_long_fw	accttgtgggtGAAtccatgccgttgaaggcgtt	charge flip, PafA site 2; together with PafA_Ala2.2_long_rv
FabD_switch1_fw	cgcctcCGTcagctcCGTttggtcccggcaaaccgcaa	charge flip FabD R1
FabD_switch1_rv	actcagcacctcggtctcgtcg	charge flip FabD R1
FabD_switch2.1_fw	gagaagctcgccCGTCGTccgccggccaGgg	charge flip FabD R2
FabD_switch2.1_rv	caacgcggtcagccgg	charge flip FabD R2

Supplementary Table 3: Protein sequences of used proteins. Differences to the wild type sequence of the respective protein is highlighted in red.

Name	Protein sequence (N → C terminus)
[Cgl]PafA3KR-His6	MSTVESALTRRIMGIETEYGLTFVDGDSKKLRPDEIARRMFRPIVEKYSSSNIFIPNGSRLYLDV<br>GSHPEYATAECDNLTQLINFEKAGDVIADRMAVDAEESLAREDIAGQVYLFKNNVDSVGNSYGCH<br>ENYLVGRSMPLKALGKRLMPFLITRQLICGAGRIHHPNPLDRGESFPLGYCISQRSDHVWEGVSS<br>ATTRSRPIINTRDEPHADSHSYRRLHVIVGDANMAEPSIALKVGSTLLVLEMIEADFGLPSLELA<br>NDIASIREISRDATGSTLLSLKDGTTMTALQIQQVVFEHASRWLEQRPEPEFSGTSNTEMARVLD<br>LWGRMLKAIESGDFSEVDTEIDWVIKKKLIDRFIQRGNLGLDDPKLAQVDLTYHDIRPGRGLFSV<br>LQSRGMIKRWTTDEAILAAVDTAPDTTRAHLRGRILKAADTLGVPVTVDWMRHKVNRPEPQSVEL<br>GDPFSAVNSEVDQLIEYMTVHAESYRSKSSVEHHHHHH
[Mtb]FabD3KR-Strep	MIALLAPGQGSQTEGMLSPWLQLPGAADQIAAWSRAADLDLARLGTTASTEEITDTAVAQPLIVA<br>ATLLAHQELARRCVLAGKDVIVAGHSVGEIAAYAIAGVIAADDAVALAATRGAEMARACATEPTG<br>MSAVLGGDETEVLSRLEQLDLVPANRNAAGQIVAAGRLTALEKLAEDPPARARVRALGVAGAFHT<br>EFMAPALDGFAAAAANIATADPTATLLSNRDGKPVTSAAAAMDTLVSQLTQPVRWDLCTATLREH<br>TVTAIVEFPPAGTLSGIAKRELRGVPARAVKSPADLDELANLWSHPQFEK
[Mtb]PanB-Strep	MAEQTIYGANTPGGSGPRTKIRTHHLQRWKADGHKWAMLTAYDYSTARIFDEAGIPVLLVGDSAA<br>NVVYGYDTTVPISIDELIPLVRGVVRGAPHALVVADLPFGSYEAGPTAALAAATRFLKDGGAHAV<br>KLEGGERVAEQIACLTAAGIPVMAHIGFTPQSVNTLGGFRVQGRGDAAEQTIADAIAVAEAGAFA<br>VVMEMVPAELATQITGKLTIPTVGIGAGPNCDGQVLVWQDMAGFSGAKTARFVKRYADVGGELRR<br>AAMQYAQEVAGGVFPADEHSFWSHPQFEK

Supplementary Table 4 Sequences and pupylation site of known good or intermediate substrates. Primary sequence ± 10 residues surrounding the central lysine (bold) were used for the creation of the Logo plot in Supplementary Figure 1c.

Substrate	Identifier	Pupylation site	Sequence around pupylation site
Mpa	Rv2115c	591	IVYIRTLVTG**K**SSSASRAIDT
PanB	Rv2225	212	PAELATQITG**K**LTIPTVGIGA
FabD	Rv2243	122	LAATRGAEMA**K**ACATEPTGMS
		173	VAAGRLTALE**K**LAEDPPAKAR
		181	LEKLAEDPPA**K**ARVRALGVAG
PckA	Rv0211	486	DYFQHWINLG**K**HADESKLPKV
IdeR	Rv2711	229	HEMAHAVKVE**K**V---------
Ino1	Rv0046c	73	KFVAAFDVDA**K**KVGFDLSDAI
Icl1	Rv0467	342	FQKELAAMGF**K**FQFITLAGFH
Log	Rv1205	74	CGGWTVGVIP**K**MLVYRELADH
RecA	Rv2737c	762	ENADVADEIE**K**KIKEKLGIGA
Kgd	Rv1248c	841	IPAGLATAVD**K**SLLARIGDAF

Supplementary references

1. Crooks, G.E., Hon, G., Chandonia, J.M. & Brenner, S.E. WebLogo: A sequence logo generator. *Genome Research* **14**, 1188-1190 (2004).
2. Erdos, G., Pajkos, M. & Dosztanyi, Z. IUPred3: prediction of protein disorder enhanced with unambiguous experimental annotation and visualization of evolutionary conservation. *Nucleic Acids Research* **49**, W297-W303 (2021).
3. Pei, J.M. & Grishin, N.V. AL2CO: calculation of positional conservation in a protein sequence alignment. *Bioinformatics* **17**, 700-712 (2001).
4. Schubert, O.T. et al. Absolute Proteome Composition and Dynamics during Dormancy and Resuscitation of Mycobacterium tuberculosis. *Cell Host & Microbe* **18**, 96-108 (2015).
5. Festa, R.A. et al. Prokaryotic ubiquitin-like protein (Pup) proteome of Mycobacterium tuberculosis. *PLoS One* **5**, e8589 (2010).
6. Watrous, J. et al. Expansion of the mycobacterial "PUPylome". *Mol Biosyst* **6**, 376-85 (2010).
7. Delley, C.L., Striebel, F., Heydenreich, F.M., Ozcelik, D. & Weber-Ban, E. Activity of the mycobacterial proteasomal ATPase Mpa is reversibly regulated by pupylation. *J Biol Chem* **287**, 7907-14 (2012).
8. Pearce, M.J. et al. Identification of substrates of the Mycobacterium tuberculosis proteasome. *EMBO J* **25**, 5423-32 (2006).
9. Guth, E., Thommen, M. & Weber-Ban, E. Mycobacterial ubiquitin-like protein ligase PafA follows a two-step reaction pathway with a phosphorylated pup intermediate. *J Biol Chem* **286**, 4412-9 (2011).
10. Poulsen, C. et al. Proteome-wide identification of mycobacterial pupylation targets. *Mol Syst Biol* **6**, 386 (2010).
11. Laederach, J.C. Doctoral Thesis, ETH Zurich (2018).
12. Regev, O., Roth, Z., Korman, M., Khalaila, I. & Gur, E. A kinetic model for the prevalence of mono- over poly-pupylation. *FEBS J* **282**, 4176-86 (2015).
13. Samanovic, M.I. et al. Proteasomal control of cytokinin synthesis protects Mycobacterium tuberculosis against nitric oxide. *Mol Cell* **57**, 984-994 (2015).
14. Muller, A.U., Leibundgut, M., Ban, N. & Weber-Ban, E. Structure and functional implications of WYL domain-containing bacterial DNA damage response regulator PafBC. *Nat Commun* **10**, 4653 (2019).
15. Elharar, Y. et al. Survival of mycobacteria depends on proteasome-mediated amino acid recycling under nutrient limitation. *EMBO J* **33**, 1802-14 (2014).
