# Supplementary material for: Electrostatic interactions guide substrate recognition of the prokaryotic ubiquitin-like protein ligase PafA
Source: Nat Commun. 2023 Aug 29;14:5266. doi: 10.1038/s41467-023-40807-8 (PMC10465538; doi:10.1038/s41467-023-40807-8)
Supplement: Supplementary file 3 — Description of additional supplementary files [file 41467_2023_40807_MOESM3_ESM.pdf]

Description of Additional Supplementary Files Document

File Name: Supplementary Data 1

Description: Two independent sets of MS data for the identification of pupylation sites in Corynebacterium glutamicum PafA.

File Name: Supplementary Data 2

Description: MS data for the identification of off-target pupylation sites in FabD-D177A as indicated in Figure 3b.
