# Supplementary material for: Electrostatic interactions guide substrate recognition of the prokaryotic ubiquitin-like protein ligase PafA
Source: Nat Commun. 2023 Aug 29;14:5266. doi: 10.1038/s41467-023-40807-8 (PMC10465538; doi:10.1038/s41467-023-40807-8)
Supplement: Supplementary file 6 — Reporting Summary [file 41467_2023_40807_MOESM6_ESM.pdf]

nature portfolio

Corresponding author(s): Eilika Weber-Ban

Last updated by author(s): Aug 03, 2023

Reporting Summary

Nature Portfolio wishes to improve the reproducibility of the work that we publish. This form provides structure for consistency and transparency in reporting. For further information on Nature Portfolio policies, see our Editorial Policies and the Editorial Policy Checklist.

Statistics

For all statistical analyses, confirm that the following items are present in the figure legend, table legend, main text, or Methods section.

n/a	Confirmed	
☐	☒	The exact sample size (*n*) for each experimental group/condition, given as a discrete number and unit of measurement
☐	☒	A statement on whether measurements were taken from distinct samples or whether the same sample was measured repeatedly
☐	☒	The statistical test(s) used AND whether they are one- or two-sided<br>*Only common tests should be described solely by name; describe more complex techniques in the Methods section.*
☒	☐	A description of all covariates tested
☒	☐	A description of any assumptions or corrections, such as tests of normality and adjustment for multiple comparisons
☐	☒	A full description of the statistical parameters including central tendency (e.g. means) or other basic estimates (e.g. regression coefficient) AND variation (e.g. standard deviation) or associated estimates of uncertainty (e.g. confidence intervals)
☐	☒	For null hypothesis testing, the test statistic (e.g. *F*, *t*, *r*) with confidence intervals, effect sizes, degrees of freedom and *P* value noted<br>*Give P values as exact values whenever suitable.*
☒	☐	For Bayesian analysis, information on the choice of priors and Markov chain Monte Carlo settings
☒	☐	For hierarchical and complex designs, identification of the appropriate level for tests and full reporting of outcomes
☐	☒	Estimates of effect sizes (e.g. Cohen's *d*, Pearson's *r*), indicating how they were calculated

Our web collection on statistics for biologists contains articles on many of the points above.

Software and code

Policy information about availability of computer code

Data collection	Kolmogorov-Smirnov test and Welch's t-test for the difference in mean protein abundance, mean protein size, mean lysine count and mean lysine density for the pupylated and non pupylated proteome was calculated with the Python implementation in the scipy.stats library v1.7.3<br>For SASA calculation the algorithm by Shrake, A. & Rupley, J.A, 1973 from the MDTraj library v1.9.3 and available protein structures (PDB identifiers are listed in Supplemental table 1) were used.<br>The script for the pupylome analysis together with the minimal data set can be found here: https://www.github.com/EWBlab/PupStatistics
Data analysis	Anaconda 3 environment with Python 3.9, GraphPad Prism 9, PyMol 2.4.1, GelAnalyzer 19.1

For manuscripts utilizing custom algorithms or software that are central to the research but not yet described in published literature, software must be made available to editors and reviewers. We strongly encourage code deposition in a community repository (e.g. GitHub). See the Nature Portfolio guidelines for submitting code & software for further information.

Data

Policy information about availability of data

All manuscripts must include a data availability statement. This statement should provide the following information, where applicable:

- Accession codes, unique identifiers, or web links for publicly available datasets
- A description of any restrictions on data availability
- For clinical datasets or third party data, please ensure that the statement adheres to our policy

All data sets are contained in the manuscript or the Supplementary material. A Source Data file has been placed alongside the manuscript. The following protein structures from the Protein Data Bank with PDB accession codes 7PXC, 1OY0, 2QC3, 4R43, 1FX7, 1GR0, 1F8I, AF-O05306-F1, 4PSK, AF-P9WIS5-F1, 2CDN, 5E0S, 5KVU, 4TVO, 4BJR, and 1UBQ were used throughout this study.

Research involving human participants, their data, or biological material

Policy information about studies with human participants or human data. See also policy information about sex, gender (identity/presentation), and sexual orientation and race, ethnicity and racism.

Reporting on sex and gender	N/A
Reporting on race, ethnicity, or other socially relevant groupings	N/A
Population characteristics	N/A
Recruitment	N/A
Ethics oversight	N/A

Note that full information on the approval of the study protocol must also be provided in the manuscript.

Field-specific reporting

Please select the one below that is the best fit for your research. If you are not sure, read the appropriate sections before making your selection.

☒ Life sciences ☐ Behavioural & social sciences ☐ Ecological, evolutionary & environmental sciences

For a reference copy of the document with all sections, see nature.com/documents/nr-reporting-summary-flat.pdf

Life sciences study design

All studies must disclose on these points even when the disclosure is negative.

Sample size	No statistical tests were used to predetermine sample sizes. In vitro assays were performed in at least three independent replicates on different days. In vivo assays were performed in at least three independent replicates on different days starting from fresh cultures. Sample size was chosen based on established practice in the field and was sufficient as the results were reproducible.
Data exclusions	No data was excluded from the analysis.
Replication	Experiments were each repeated three or more times. Results were reproducible.
Randomization	Not applicable to our study, since no treatment groups were handled in the experiments
Blinding	Not applicable to our study. No populations were preassigned to groups so blinding was not relevant. Handled samples needed to be identifiable throughout the study.

Reporting for specific materials, systems and methods

We require information from authors about some types of materials, experimental systems and methods used in many studies. Here, indicate whether each material, system or method listed is relevant to your study. If you are not sure if a list item applies to your research, read the appropriate section before selecting a response.

Materials & experimental systems

n/a	Involved in the study	
☐	☒	Antibodies
☒	☐	Eukaryotic cell lines
☒	☐	Palaeontology and archaeology
☒	☐	Animals and other organisms
☒	☐	Clinical data
☒	☐	Dual use research of concern
☒	☐	Plants

Methods

n/a	Involved in the study	
☒	☐	ChIP-seq
☒	☐	Flow cytometry
☒	☐	MRI-based neuroimaging

Antibodies

Antibodies used	anti-RpoB (E. coli) monoclonal (mouse): BioLegend, clone #8RB13, Cat. #663903<br>anti-Pup (M. tuberculosis) polyclonal (rabbit): described in Striebel, F. et al., 2009<br>anti-rabbit IgG HRP polyclonal (goat): Abcam, Cat. #ab6721<br>anti-mouse IgG HRP polyclonal (goat): Promega, Ca. #W4021
Validation	anti-RpoB (E. coli) was validated by the manufacturer via western blot analysis as outlined on the website.<br>Application reference in Actinobacteria: Stalder, ES. et al. 2011. Protein Expr. Purif. 77(1):26-33. (Epitope, ELISA, IP, WB)<br>anti-Pup (M. tuberculosis) was validated by us via western blot analysis published in Striebel, F. et al., 2009<br>anti-rabbit IgG HRP and anti-mouse IgG HRP were validated by the manufactures via western blot analysis as outlined on their websites.
